# Frequent Mental Distress Among Adults, by Disability Status, Disability Type, and Selected Characteristics — United States, 2018

**DOI:** 10.15585/mmwr.mm6936a2

**Published:** 2020-09-11

**Authors:** Robyn A. Cree, Catherine A. Okoro, Matthew M. Zack, Eric Carbone

**Affiliations:** ^1^Division of Human Development and Disability, National Center on Birth Defects and Developmental Disabilities, CDC; ^2^Division of Population Health, National Center for Chronic Disease Prevention and Health Promotion, CDC.

Frequent mental distress, defined as 14 or more self-reported mentally unhealthy days in the past 30 days,* is associated with adverse health behaviors, increased use of health services, mental disorders (e.g., diagnosis of major depressive disorder), chronic diseases, and functional limitations (*1*). Adults with disabilities more often report depression and anxiety (*2*), reduced health care access (*3*), and health-related risk behaviors (*4*) than do adults without disabilities. CDC analyzed 2018 Behavioral Risk Factor Surveillance System (BRFSS) data to compare the prevalence of frequent mental distress among adults with disabilities with that among adults without disabilities and to identify factors associated with mental distress among those with disabilities. Nationwide, an estimated 17.4 million adults with disabilities reported frequent mental distress; the prevalence of reported mental distress among those with disabilities (32.9%) was 4.6 times that of those without disabilities (7.2%). Among adults with disabilities, those with both cognitive and mobility disabilities most frequently reported mental distress (55.6%). Adults with disabilities who reported adverse health-related characteristics (e.g., cigarette smoking, physical inactivity, insufficient sleep, obesity, or depressive disorders) or an unmet health care need because of cost also reported experiencing more mental distress than did those with disabilities who did not have these characteristics. Adults living below the federal poverty level reported mental distress 70% more often than did adults in higher income households. Among states, age-adjusted prevalence of mental distress among adults with disabilities ranged from 25.2% (Alaska) to 42.9% (New Hampshire). Understanding the prevalence of mental distress among adults with disabilities could help health care providers, public health professionals, and policy makers target interventions and inform programs and policies to ensure receipt of mental health screening, care, and support services to reduce mental distress among adults with disabilities.

BRFSS is an annual, landline and cellular telephone–based self-reported survey of noninstitutionalized U.S. adults aged ≥18 years.^†^ In 2018, the BRFSS unweighted sample size was 430,949. The combined (landline and cellular telephone) median response rate among the 50 states and the District of Columbia in 2018 was 49.9% (range = 38.8%–67.2%).^§^ Adults were considered to have a disability if they reported having one or more of six disability types: hearing, vision, cognition, mobility, self-care, or independent living.^¶^^,^** Mutually exclusive disability categories were created for each disability type and for adults reporting more than one disability. The latter were further categorized into four groups, based on cognition or mobility, two of the most prevalent disability types: cognition-only, mobility-only, both, or neither. Adults were considered to have frequent mental distress if they reported 14 or more days in response to the question “Now thinking about your mental health, which includes stress, depression, and problems with emotions, for how many days during the past 30 days was your mental health not good?”

CDC compared the prevalence of mental distress among adults with and without disabilities by disability type and selected demographic characteristics that included sex, age, race/ethnicity, veteran status, marital status, employment status, sexual identity,^††^ federal poverty level, and urban/rural designation.^§§^ CDC calculated age-standardized^¶¶^ prevalences, 95% confidence intervals, and age-adjusted*** prevalence ratios (PRs) to compare mental distress among adults with disabilities with that among those without disabilities. Among adults with disabilities, CDC compared age-standardized prevalences of mental distress by disability type and health-related characteristics, including health care access,^†††^ health-related behaviors,^§§§^ obesity,^¶¶¶^ and a diagnosed depressive disorder.**** Age-standardized prevalence estimates and age-adjusted PRs of mental distress were calculated among adults with disabilities by state of residence. Missing responses to questions about disability and mental distress were excluded from analyses, resulting in a total unweighted analytic sample size of 404,973. For all comparisons, statistical significance at a level of α = 0.05 was determined using a two-sided t-test in SAS-callable SUDAAN (version 11.0.1; RTI International).

Overall, 26.2% of U.S. adults who responded to questions about disability and mental distress reported having a disability. Nearly one third of adults with disabilities (32.9%) reported experiencing frequent mental distress, compared with 7.2% of adults without disabilities (PR = 4.6) (Table 1). Frequent mental distress was reported by 55.6% of those with disability in both mobility and cognition, 8.8 times that reported among those without disabilities. Demographic differences in PRs of mental distress were generally similar among adults with and without disabilities, except for veteran and employment status. Mental distress was more commonly reported among females and persons who were unmarried; unemployed; identified as lesbian or gay, bisexual, or something else; and lived in lower-income households compared with males and those who were married, employed, identified as straight or not gay, and lived in higher-income households. Persons identifying as non-Hispanic Asian, Hispanic, and middle-aged or older reported mental distress less often than did those who identified as non-Hispanic white, and who were younger. Among adults without disabilities, both veterans and retirees were 20% less likely to report mental distress than were nonveterans and adults who were employed; no differences were found by veteran and employment status for adults with disabilities.

**TABLE 1:** Age-adjusted prevalence of frequent mental distress, by disability status and types,* and prevalence ratios, by selected demographic characteristics — Behavioral Risk Factor Surveillance System, United States, 2018

Characteristic	Adults with a disability (n = 119,196) % (95% CI)^†^	PR (95% CI)^§^	Adults without a disability (n = 285,777) % (95% CI)^†^	PR (95% CI)^§^
**Overall^¶^**	**32.9 (32.2–33.6)**	**4.6 (4.5–4.8)**	**7.2 (7.0–7.4)**	**—**
**Disability type and number of disabilities (1 versus >1)^¶^**				
1 disability type (no.**)				
Total (n = 67,116)	23.9 (23.1–24.8)	3.2 (3.0–3.3)	—	—
Cognition only (14,776)	35.7 (34.2–37.1)	4.8 (4.6–5.1)	—	—
Independent living only (2,739)	31.6 (28.2–35.1)	4.5 (4.0–5.0)	—	—
Mobility only (25,612)	17.3 (15.5–19.3)	2.4 (2.2–2.7)	—	—
Self-care only (652)	12.6 (7.9–19.4)	1.9 (1.2–2.9)	—	—
Vision only (5,994)	11.6 (10.0–13.4)	1.6 (1.4–1.9)	—	—
Hearing only (17,343)	11.4 (9.1–14.1)	1.4 (1.2–1.6)	—	—
>1 disability type (no.**)				
Total (n = 52,080)	45.6 (44.4–46.7)	6.5 (6.3–6.8)	—	—
Mobility and cognition (18,563)	55.6 (53.8–57.5)	8.8 (8.4–9.1)	—	—
Cognition without mobility (8,389)	51.6 (49.6–53.7)	7.1 (6.8–7.5)	—	—
Mobility without cognition (21,999)	26.0 (23.6–28.5)	4.1 (3.8–4.4)	—	—
Neither cognition nor mobility (3,129)	22.5 (18.5–27.1)	3.0 (2.5–3.5)	—	—
**Demographic characteristic**				
**Sex**				
Male	28.0 (27.0–29.0)	Reference	6.0 (5.7–6.3)	Reference
Female	37.0 (36.1–38.0)	1.3 (1.2–1.3)	8.4 (8.1–8.7)	1.4 (1.3–1.5)
**Age group, yrs^††^**				
18–44	40.4 (39.2–41.6)	Reference	9.4 (9.0–9.7)	Reference
45–64	30.6 (29.8–31.5)	0.8 (0.7–0.8)	5.5 (5.2–5.8)	0.6 (0.6–0.6)
≥65	13.5 (12.8–14.1)	0.3 (0.3–0.4)	3.5 (3.2–3.8)	0.4 (0.3–0.4)
**Race/Ethnicity**				
White, non-Hispanic	36.5 (35.6–37.3)	Reference	7.7 (7.5–7.9)	Reference
Black, non-Hispanic	30.8 (28.8–32.8)	0.9 (0.8–1.0)	7.3 (6.8–8.0)	1.0 (0.9–1.0)
Asian, non-Hispanic	23.4 (19.0–28.4)	0.6 (0.5–0.8)	4.8 (3.9–6.0)	0.6 (0.5–0.7)
AI/AN, non-Hispanic	34.1 (29.8–38.6)	1.0 (0.9–1.2)	8.4 (6.6–10.7)	1.1 (0.8–1.4)
Hispanic	25.7 (23.9–27.5)	0.7 (0.7–0.8)	6.5 (5.9–7.2)	0.8 (0.7–0.9)
Other race/Multiracial	40.5 (37.5–43.6)	1.1 (1.1–1.2)	8.6 (7.6–9.6)	1.1 (1.0–1.2)
**Veteran status**				
Yes	34.8 (32.3–37.4)	1.0 (0.9–1.0)	6.1 (5.4–6.8)	0.8 (0.7–0.9)
No	32.8 (32.1–33.5)	Reference	7.3 (7.1–7.5)	Reference
**Marital status**				
Married/Unmarried couple	28.5 (27.4–29.5)	Reference	5.8 (5.5–6.0)	Reference
Divorced/Separated	39.0 (37.1–41.0)	1.5 (1.4–1.5)	9.0 (8.3–9.7)	1.6 (1.4–1.7)
Widowed	39.2 (33.8–44.9)	1.3 (1.2–1.4)	12.2 (8.8–16.6)	1.9 (1.6–2.1)
Never married	34.9 (33.6–36.2)	1.2 (1.2–1.3)	9.2 (8.6–9.7)	1.6 (1.5–1.7)
**Employment status**				
Employed	26.6 (25.7–27.6)	Reference	6.7 (6.5–7.0)	Reference
Unemployed	40.8 (38.1–43.6)	1.5 (1.4–1.6)	10.8 (9.7–12.1)	1.5 (1.4–1.7)
Homemaker or student	30.3 (28.2–32.5)	1.2 (1.1–1.2)	8.3 (7.7–9.0)	1.2 (1.1–1.3)
Retired	31.2 (24.8–38.4)	1.0 (0.9–1.1)	3.4 (2.3–5.1)	0.8 (0.7–0.9)
Unable to work	45.0 (43.3–46.6)	1.8 (1.7–1.9)	16.9 (13.7–20.5)	2.6 (2.2–3.0)
**Sexual identity^§§^**				
Straight (not gay)	31.0 (30.0–32.1)	Reference	6.9 (6.6–7.2)	Reference
Lesbian or gay	41.6 (36.3–47.2)	1.3 (1.2–1.5)	10.9 (8.5–13.9)	1.6 (1.2–2.1)
Bisexual	48.2 (43.8–52.6)	1.6 (1.5–1.8)	13.9 (12.1–15.8)	2.2 (1.9–2.5)
Something else	43.1 (37.0–49.4)	1.4 (1.2–1.6)	13.7 (10.4–17.9)	2.0 (1.5–2.6)
**Federal poverty level, % above or below**				
≥400% (higher income)	25.6 (23.8–27.4)	Reference	5.6 (5.3–5.9)	Reference
200% to <400%	28.9 (27.2–30.6)	1.2 (1.1–1.3)	7.9 (7.4–8.4)	1.4 (1.3–1.5)
100% to <200%	36.3 (34.9–37.8)	1.6 (1.5–1.7)	9.0 (8.5–9.6)	1.6 (1.5–1.8)
<100% (lower income)	38.6 (37.1–40.1)	1.7 (1.5–1.8)	10.2 (9.3–11.1)	1.7 (1.6–1.9)
Unknown	31.5 (29.9–33.2)	1.3 (1.2–1.4)	6.7 (6.2–7.2)	1.2 (1.1–1.3)
**Urban/Rural designation^¶¶^**				
Large central metro	30.0 (28.5–31.6)	Reference	7.1 (6.6–7.5)	Reference
Large fringe metro	33.5 (31.9–35.1)	1.1 (1.0–1.2)	6.8 (6.4–7.2)	1.0 (0.9–1.1)
Small/Medium metro	34.4 (33.3–35.6)	1.1 (1.1–1.2)	7.6 (7.3–7.9)	1.1 (1.0–1.2)
Nonmetropolitan	34.0 (32.7–35.3)	1.1 (1.1–1.2)	7.5 (7.0–8.0)	1.1 (1.0–1.2)

**Abbreviations:** AI/AN = American Indian/Alaska Native; CI = confidence interval; PR = prevalence ratio.

* Adults were considered to have a disability if they reported having one or more of the following six disability types: hearing, vision, cognition, mobility, self-care, or independent living. Mutually exclusive disability categories were created for each of the six disability types and for persons reporting >1 disability. Respondents reporting >1 disability were further categorized into four groups, based on whether they reported the most commonly reported disability types, cognition or mobility (cognition-only, mobility-only, both, or neither).

^†^ Percentages are weighted and standardized based on the population breakdown for the following age groups according to the 2000 U.S. Census: 18–44, 45–64, and ≥65 years. https://www.cdc.gov/nchs/data/statnt/statnt20.pdf.

^§^ Models estimating PRs are adjusted for age group (18–44, 45–64, and ≥65 years).

^¶^ No disability is the reference category for overall and PRs for disability types. PRs for the overall and disability type categories were generated from three models using three categorizations of disability: two-level categorization (any disability, no disability); 8-level categorization (cognition only, independent living only, mobility only, self-care only, vision only, and hearing only, >1 disability, no disability); and 6-level categorization (mobility + cognition, cognition no mobility, mobility no cognition, neither cognition or mobility, 1 disability, no disability).

** Unweighted sample size.

^††^ Percentages and PRs are not standardized or adjusted.

^§§^ Optional module asked in 29 states: Arizona, Connecticut, Delaware, Florida, Hawaii, Idaho, Illinois, Kansas, Louisiana, Maryland, Minnesota, Mississippi, Missouri, Montana, Nevada, New York, North Carolina, Ohio, Oklahoma, Pennsylvania, Rhode Island, South Carolina, Tennessee, Texas, Vermont, Virginia, Washington, West Virginia, and Wisconsin.

^¶¶^ Urban/rural designations based on 2013 Urban-Rural Classification Scheme for Counties. https://www.cdc.gov/nchs/data_access/urban_rural.htm.

Among adults with disabilities, those who reported adverse health-related behaviors or conditions (i.e., cigarette smoking, insufficient sleep, physical inactivity, obesity, and diagnosed depressive disorder) or an unmet health care need because of cost more often had frequent mental distress than did those without these characteristics (Table 2). In general, patterns were similar across disability types. All health-related factors were associated with mental distress for adults without disabilities (Supplementary Table 1, https://stacks.cdc.gov/view/cdc/92748). Among adults with disabilities, the highest prevalences of frequent mental distress were in New Hampshire (42.9%), South Carolina (39.2%), and Maine (38.7%); the median prevalence (32.5%) was in Louisiana; and the lowest prevalences were in Alaska (25.2%), Hawaii (26.7%), and Illinois (26.9%) (Figure) (Supplementary Table 2, https://stacks.cdc.gov/view/cdc/92748). Adults with disabilities in Minnesota, Kansas, Georgia, Iowa, and Delaware were 5.4–5.7 times more likely to report frequent mental distress than were adults without disabilities, whereas in Illinois and West Virginia, the PRs were 3.5 and 3.6, respectively (Figure) (Supplementary Table 2, https://stacks.cdc.gov/view/cdc/92748).

**TABLE 2:** Age-adjusted prevalence of frequent mental distress, by disability type,* and selected health-related characteristics among adults with disabilities — Behavioral Risk Factor Surveillance System, United States, 2018

Characteristic	Disability % (95% CI)^†^								P-value^§^
	Cognition only (n = 14,776)	Independent living only (n = 2,739)	Mobility only (n = 25,612)	Self-care only (n = 652)	Vision only (n = 5,994)	Hearing only (n = 17,343)	>1 disability type (n = 52,080)	All (n = 119,196)	
**Health care factor**									
**Health insurance coverage**									
Yes	36.4 (34.8–38.0)	31.6 (28.0–35.4)	17.1 (15.1–19.3)	13.8 (9.1–20.4)	11.7 (9.8–13.9)	10.6 (8.2–13.8)	45.6 (44.3–46.9)	33.1 (32.3–33.8)	0.9
No	31.0 (27.8–34.4)	28.4 (21.4–36.7)	18.1 (14.0–23.1)	12.1 (5.1–25.9)^¶^	11.5 (8.4–15.5)	12.6 (8.7–17.8)	48.1 (45.1–51.2)	33.2 (31.3–35.1)	
**Usual health care provider**									
Yes	35.8 (34.1–37.6)	29.6 (25.8–33.6)	17.3 (15.2–19.7)	10.4 (5.8–18.1)	12.6 (10.2–15.5)	12.2 (8.9–16.5)	45.2 (43.8–46.6)	33.2 (32.3–34.1)	0.8
No	35.9 (32.8–39.1)	35.4 (28.3–43.1)	17.9 (14.0–22.7)	19.8 (10.7–33.6)	10.8 (8.1–14.3)	10.6 (7.8–14.2)	47.9 (45.4–50.4)	33.0 (31.5–34.5)	
**Unmet health care need because of cost during past 12 mos**									
Yes	43.1 (39.4–46.8)	39.6 (32.7–46.8)	24.9 (21.0–29.4)	22.7 (12.3–38.1)	17.9 (13.7–23.1)	15.4 (11.9–19.7)	53.2 (51.2–55.2)	43.0 (41.6–44.5)	<0.001
No	33.3 (31.7–35.0)	28.6 (24.8–32.7)	15.0 (13.0–17.2)	10.3 (6.4–16.2)	9.6 (7.9–11.7)	11.0 (8.3–14.4)	42.1 (40.7–43.6)	29.4 (28.5–30.2)	
**Routine check-up within past 12 mos**									
Yes	35.9 (34.2–37.7)	30.7 (26.9–34.8)	17.2 (15.2–19.5)	12.1 (7.0–19.9)	12.0 (9.9–14.5)	11.5 (8.4–15.4)	44.9 (43.5–46.3)	32.9 (32.0–33.8)	0.6
No	33.5 (30.9–36.2)	33.6 (27.1–40.8)	17.1 (13.6–21.4)	13.1 (5.8–26.9)^¶^	10.4 (8.1–13.3)	11.6 (8.5–15.7)	48.6 (46.3–51.0)	32.5 (31.2–33.8)	
**Health-related behaviors and obesity**									
**Binge drinking**									
Yes	35.4 (32.1–38.9)	31.3 (25.1–38.2)	18.5 (14.3–23.5)	18.8 (9.4–34.2)^¶^	10.4 (7.4–14.6)	13.0 (9.3–17.8)	49.7 (46.8–52.6)	34.4 (32.7–36.2)	0.06
No	35.4 (33.8–37.1)	31.8 (28.0–35.9)	17.3 (15.3–19.6)	11.6 (7.0–18.8)	12.0 (10.0–14.4)	10.8 (8.1–14.2)	44.9 (43.6–46.2)	32.6 (31.8–33.4)	
**Cigarette smoking status**									
Current smoker	42.6 (39.7–45.6)	41.3 (34.8–48.2)	25.9 (22.0–30.2)	25.3 (14.0–41.2)	15.0 (11.9–18.7)	14.6 (11.0–19.1)	54.6 (52.7–56.5)	43.4 (42.1–44.7)	<0.001
Nonsmoker (former/never)	33.4 (31.7–35.1)	27.4 (23.7–31.5)	14.5 (12.5–16.7)	10.0 (5.7–16.8)	10.8 (8.9–13.0)	10.8 (8.1–14.2)	41.3 (39.8–42.8)	29.0 (28.1–29.8)	
**Physical inactivity**									
Yes	39.3 (36.4–42.3)	29.1 (23.0–36.0)	19.6 (16.6–23.1)	10.5 (6.5–16.5)	12.8 (9.7–16.7)	10.9 (7.6–15.3)	49.8 (48.0–51.6)	37.8 (36.5–39.2)	<0.001
No	34.4 (32.7–36.1)	31.8 (28.0–35.8)	15.9 (13.8–18.3)	17.0 (10.8–25.9)	11.2 (9.3–13.3)	11.5 (8.9–14.8)	42.0 (40.5–43.5)	29.9 (29.0–30.7)	
Obesity**									
Yes	38.0 (35.3–40.7)	32.8 (26.4–39.9)	18.7 (15.8–22.0)	12.0 (6.0–22.5)^¶^	13.1 (10.4–16.4)	12.3 (7.7–19.1)	47.9 (46.2–49.6)	35.0 (33.9–36.2)	<0.001^††^
No	35.1 (33.3–37.0)	33.3 (29.2–37.6)	16.2 (13.8–18.8)	11.8 (6.3–21.1)^¶^	11.4 (9.5–13.6)	10.6 (8.5–13.0)	44.8 (43.1–46.4)	32.3 (31.4–33.3)	
Unknown (n = 25,048)	32.3 (26.2–39.0)	12.8 (8.1–19.5)	16.6 (11.8–22.9)	30.0 (15.0–51.0)^¶^	9.5 (4.0–20.9)^§^	13.2 (6.3–25.7)^§^	39.9 (35.4–44.5)	27.4 (24.8–30.1)	
**Insufficient sleep^§§^**									
Yes	41.7 (39.4–44.0)	36.6 (31.2–42.3)	22.0 (19.1–25.2)	24.7 (15.6–36.6)	14.7 (11.9–18.1)	15.6 (11.9–20.2)	51.2 (49.7–52.7)	39.4 (38.4–40.4)	<0.001
No	30.3 (28.4–32.2)	27.7 (23.5–32.4)	13.4 (11.3–15.8)	7.2 (4.2–12.0)	9.1 (7.4–11.2)	7.6 (5.3–10.6)	38.6 (36.8–40.5)	26.3 (25.3–27.3)	
**Mental health**									
**Diagnosed depressive disorder^¶¶^**									
Yes	50.0 (47.8–52.1)	49.1 (43.5–54.8)	38.4 (34.5–42.5)	27.8 (17.0–41.9)	29.1 (22.6–36.7)	21.7 (16.2–28.5)	62.8 (61.5–64.2)	54.3 (53.3–55.4)	<0.001
No	21.9 (20.1–23.9)	16.1 (12.8–20.0)	10.1 (8.4–12.2)	9.7 (5.2–17.2)^¶^	7.9 (6.6–9.4)	9.5 (7.1–12.6)	23.7 (21.9–25.6)	16.7 (15.9–17.6)	

**Abbreviation:** CI = confidence interval.

* Mutually exclusive disability categories were created for each of the six disability types and for adults reporting two or more disabilities.

^†^ Percentages are weighted.

^§^ Two-sided p-value from the t-distribution comparing prevalence of frequent mental distress by whether or not the person had the health-related characteristic.

^¶^ Estimate is unstable (relative standard error = 30%–41%).

** Obesity was defined as body mass index ≥30.0 kg/m^2^.

^††^ Comparing yes versus no.

^§§^ Insufficient sleep is defined as getting <7 hours of sleep per 24-hour period on average per night.

^¶¶^ Includes depression, major depression, dysthymia, and minor depression.

**FIGURE:**
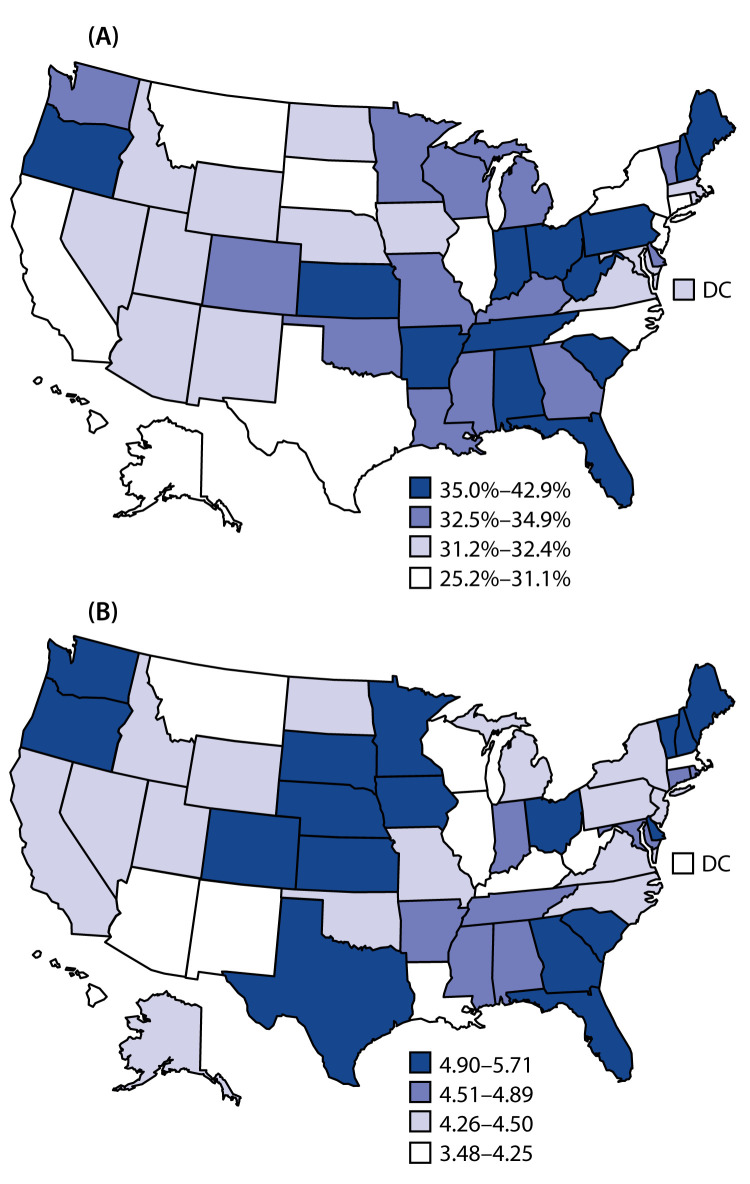
Age-adjusted prevalence* of frequent mental distress among adults with disabilities (A) and prevalence ratios of frequent mental distress between adults with and without disabilities (B), by geographic area — Behavioral Risk Factor Surveillance System, United States, 2018 **Abbreviation:** DC = District of Columbia. * Standardized to the 2000 U.S. projected population.

## Discussion

In 2018, an estimated 17.4 million U.S. adults with disabilities reported frequent mental distress across a range of demographic characteristics (including poverty and marital status), 4.6 times as often than did adults without disabilities. Having a diagnosed depressive disorder was associated with experiencing frequent mental distress, with approximately one half of adults with disabilities and a diagnosed depressive disorder reporting distress. One in six adults with disabilities who did not have a diagnosed depressive disorder reported frequent mental distress, possibly representing adults with undiagnosed mental disorders. Health care providers caring for adults with disabilities might focus on the primary disability but miss opportunities to identify and treat co-occurring mental health conditions (*5*). Furthermore, symptoms associated with some physical disabilities and chronic conditions, as well as overall level of functional impairment, might be exacerbated by mental distress and might improve with mental health treatment (*6*). To ensure recommended clinical management and referral, providers could consider screening their clients for mental health symptoms, even if mental health concerns are unrelated to the primary condition for which adults are being seen. To promote overall well-being, health care providers and public health professionals can also focus on promoting healthy lifestyles, such as maintaining a healthy weight, meeting physical activity recommendations, quitting smoking, and getting sufficient sleep,^††††^ given that these findings indicate unhealthy lifestyles are associated with mental distress.

In one 6-year longitudinal study, increases in social support were associated with decreases in depressive symptoms among adults with physical disabilities (*7*). Adults with disabilities might have fewer opportunities for high-quality social engagement because of physical limitations (*8*) or reduced ability to communicate (*9*), placing them at increased risk for experiencing mental distress. The findings of reduced mental distress among adults with disabilities who are married and employed, two factors known to correlate with social ties and support (*10*), suggest that programs aimed at increasing social connectedness might help reduce the large disparity in mental distress between adults with and without disabilities.

Because health care access concerns are prevalent among adults with disabilities (*3*), the finding that adults with a cost-related unmet health care need during the past 12 months more often reported mental distress is particularly concerning. Policies, such as the Affordable Care Act, put into place to improve health care access among adults with disabilities (particularly those living in lower-income households) might help address disparities in mental distress.

The findings in this report are subject to at least four limitations. First, causality cannot be inferred from data in this cross-sectional survey; disability or adverse health-related behaviors might cause mental distress, and such distress might worsen disability or increase risk. Second, social desirability bias can result in underreporting of mental health symptoms in survey data. Third, prevalence of mental distress might be underestimated if adults with severe functional and cognitive disabilities (with potentially higher distress) are underrepresented in BRFSS data. Finally, disability categories captured in BRFSS are broad, and the primary disabling condition was unknown. Considering that 4.6% of the noninstitutionalized U.S. population had a serious mental illness in 2018,^§§§§^ diagnosed mental disorders not assessed in BRFSS might explain in part the high prevalence of frequent mental distress among adults reporting cognitive disabilities.

This report highlights disparities in prevalence of frequent mental distress by disability status, disability type, and several demographic and health-related risk factors associated with mental and physical health. Public health professionals, policy makers, and health care providers can consider recommending strategies that increase social cohesion, encourage community participation, and improve access to quality mental health screening and care, as well as promoting healthy lifestyle recommendations and inclusion in evidence-based programs to address disparities in mental distress. Increasing provider awareness of the importance of mental health screening could help improve identification and treatment of co-occurring mental health conditions, especially among adults with cognitive and mobility disabilities who are approximately nine times as likely to have frequent mental distress as are adults without disabilities. Future work to better understand mental distress among adults with disabilities could help target interventions, whether as stand-alone approaches or components of existing disease prevention and health promotion strategies.

**Summary**

**What is already known about this topic?**

Adults with disabilities, compared with those without disabilities, experience more mental distress and are more likely to experience factors associated with a higher occurrence of mental disorders, including poverty and limited heath care access.

**What is added by this report?**

Nationwide, an estimated 17.4 million adults with disabilities experience frequent mental distress 4.6 times as often than do adults without disabilities. Adults living below the federal poverty level report mental distress 70% more often than do adults in higher income households.

**What are the implications for public health practice?**

Targeted interventions and programs and policies that ensure receipt of mental health screening, care, and support services could help reduce mental distress among adults with disabilities.

## Footnotes

* https://www.cdc.gov/hrqol/pdfs/mhd.pdf.

† https://www.cdc.gov/brfss/.

§ https://www.cdc.gov/brfss/annual_data/2018/pdf/2018-sdqr-508.pdf.

¶ Based on Section 4302 of the Affordable Care Act, the U.S. Department of Health and Human Services issued data collection standard guidance to include a standard set of disability identifiers in all national population health surveys. https://aspe.hhs.gov/datacncl/standards/aca/4302/index.pdf.

** The interviewer first reads a preamble to the telephone survey respondent (“The following questions are about health problems or impairments you may have. Some adults who are deaf or have serious difficulty hearing may or may not use equipment to communicate by phone.”), followed by the six specific disability type questions. The six questions are “Are you deaf or do you have serious difficulty hearing?” (hearing); “Are you blind or do you have serious difficulty seeing, even when wearing glasses?” (vision); “Because of a physical, mental, or emotional condition, do you have serious difficulty concentrating, remembering, or making decisions?” (cognition); “Do you have serious difficulty walking or climbing stairs?” (mobility); “Do you have difficulty dressing or bathing?” (self-care); and “Because of a physical, mental, or emotional condition, do you have difficulty doing errands alone such as visiting a doctor’s office or shopping?” (independent living).

†† Optional module asked in 29 states: Arizona, Connecticut, Delaware, Florida, Hawaii, Idaho, Illinois, Kansas, Louisiana, Maryland, Minnesota, Mississippi, Missouri, Montana, Nevada, New York, North Carolina, Ohio, Oklahoma, Pennsylvania, Rhode Island, South Carolina, Tennessee, Texas, Vermont, Virginia, Washington, West Virginia, and Wisconsin.

§§ Urban/rural designation based on 2013 Urban-Rural Classification Scheme for Counties. https://www.cdc.gov/nchs/data_access/urban_rural.htm.

¶¶ Based on the population breakdown for the following age groups according to the 2000 U.S. Census: 18–44, 45–64, and ≥65 years. https://www.cdc.gov/nchs/data/statnt/statnt20.pdf.

*** Models estimating PRs are adjusted for age (18–44, 45–64, and ≥65 years).

††† Health insurance coverage was ascertained by a “yes” response to the question “Do you have any kind of health care coverage, including health insurance, prepaid plans such as health maintenance organizations, government plans such as Medicare, or Indian Health Service?” Having a usual health care provider was assessed first with the question “Do you have one person you think of as your personal doctor or health care provider?” Adults who responded “no” were asked the question “Is there more than one, or is there no person who you think of as your personal doctor or health care provider?” Responses for having a usual health care provider were dichotomized into one or more and none. Unmet health care need because of cost was ascertained by a “yes” response to the question “Was there a time in the past 12 months when you needed to see a doctor but could not because of cost?” Receipt of a routine check-up was assessed with the question “About how long has it been since you last visited a doctor for a routine checkup? A routine checkup is a general physical exam, not an exam for a specific injury, illness, or condition.” Responses for having had a routine checkup within the preceding 12 months were dichotomized into within the past year or not within the past year.

§§§ Binge drinking was based on a response of one or more to the question “Considering all types of alcoholic beverages, how many times during the past 30 days did you have 5 or more drinks for men or 4 or more drinks for women on an occasion?” Cigarette smoking status was determined by a response of “Every day” or “Some days” to the question “Do you now smoke cigarettes every day, some days, or not at all?” Physical inactivity was ascertained by a response of “no” to the question “During the past month, other than your regular job, did you participate in any physical activities or exercises such as running, calisthenics, golf, gardening, or walking for exercise?” Insufficient sleep was defined as getting less than 7 hours of sleep in response to the question “On average, how many hours of sleep do you get in a 24-hour period?”

¶¶¶ Obesity was defined as body mass index ≥30.0 kg/m^2^.

**** Diagnosed depressive disorder was ascertained by a “yes” to having a depressive disorder (including depression, major depression, dysthymia, and minor depression) in response the question “Has a doctor, nurse, or other health professional ever told you that you have any of the following?”

†††† Defined as ≥7 hours in a 24-hour period. https://www.cdc.gov/sleep/about_sleep/how_much_sleep.html.

§§§§ https://www.samhsa.gov/data/report/2018-nsduh-detailed-tables.
